# SGLT2 inhibition with empagliflozin improves coronary microvascular function and cardiac contractility in prediabetic ob/ob^−/−^ mice

**DOI:** 10.1186/s12933-019-0820-6

**Published:** 2019-02-07

**Authors:** Damilola D. Adingupu, Sven O. Göpel, Julia Grönros, Margareta Behrendt, Matus Sotak, Tasso Miliotis, Ulrika Dahlqvist, Li-Ming Gan, Ann-Cathrine Jönsson-Rylander

**Affiliations:** 1 Bioscience, Cardiovascular, Renal and Metabolic Diseases, IMED Biotech Unit, AstraZeneca Gothenburg, Pepparedsleden 1, Mölndal, 431 83 Gothenburg, Sweden; 2 Translational Science, Cardiovascular, Renal and Metabolic Diseases, IMED Biotech Unit, AstraZeneca Gothenburg, Gothenburg, Sweden; 3 Early Clinical Development, Cardiovascular, Renal and Metabolic Diseases, IMED Biotech Unit, AstraZeneca Gothenburg, Gothenburg, Sweden; 4 Department of Molecular and Clinical Medicine, Institute of Medicine, Sahlgrenska Academy at the University of Gothenburg, Gothenburg, Sweden; 5 Department of Cardiology, Sahlgrenska University Hospital, Gothenburg, Sweden

**Keywords:** Coronary, Endothelial, Microvascular, Prediabetes, SGLT2

## Abstract

**Background:**

Sodium-glucose cotransporter 2 inhibitors (SGLT2i) is the first class of anti-diabetes treatment that reduces mortality and risk for hospitalization due to heart failure. In clinical studies it has been shown that SGLT2i’s promote a general shift to fasting state metabolism characterized by reduced body weight and blood glucose, increase in glucagon/insulin ratio and modest increase in blood ketone levels. Therefore, we investigated the connection between metabolic changes and cardiovascular function in the ob/ob^−/−^ mice; a rodent model of early diabetes with specific focus on coronary microvascular function. Due to leptin deficiency these mice develop metabolic syndrome/diabetes and hepatic steatosis. They also develop cardiac contractile and microvascular dysfunction and are thus a promising model for translational studies of cardiometabolic diseases. We investigated whether this mouse model responded in a human-like manner to empagliflozin treatment in terms of metabolic parameters and tested the hypothesis that it could exert direct effects on coronary microvascular function and contractile performance.

**Methods:**

Lean, ob/ob^−/−^ untreated and ob/ob^−/−^ treated with SGLT2i were followed for 10 weeks. Coronary flow velocity reserve (CFVR) and fractional area change (FAC) were monitored with non-invasive Doppler ultrasound imaging. Food intake, urinary glucose excursion and glucose control via HbA1c measurements were followed throughout the study. Liver steatosis was assessed by histology and metabolic parameters determined at the end of the study.

**Results:**

Sodium-glucose cotransporter 2 inhibitors treatment of ob/ob^−/−^ animals resulted in a switch to a more catabolic state as observed in clinical studies: blood cholesterol and HbA1c were decreased whereas glucagon/insulin ratio and ketone levels were increased. SGLT2i treatment reduced liver triglyceride, steatosis and alanine aminotransferase, an indicator for liver dysfunction. l-Arginine/ADMA ratio, a marker for endothelial function was increased. SGLT2i treatment improved both cardiac contractile function and coronary microvascular function as indicated by improvement of FAC and CFVR, respectively.

**Conclusions:**

Sodium-glucose cotransporter 2 inhibitors treatment of ob/ob^−/−^ mice mimics major clinical findings regarding metabolism and cardiovascular improvements and is thus a useful translational model. We demonstrate that SGLT2 inhibition improves coronary microvascular function and contractile performance, two measures with strong predictive values in humans for CV outcome, alongside with the known metabolic changes in a preclinical model for prediabetes and heart failure.

## Background

The risk of cardiovascular (CV) disease is increased in type 2 diabetes mellitus (T2DM), and it is recognized that microvascular and macrovascular complications occur in individuals with T2DM [1]. Further, individuals with prediabetes are at higher risk of suffering from CV events [2]. Current evidence also shows that there is a bi-directional link between fatty liver and CV disease [3]. Antidiabetic treatments that are both effective against underlying pathology in T2DM as well as associated CV complications including fatty liver disease will be beneficial for the patients in improving prognosis [4]. In addition, the recent clinical trials, EMPA-REG OUTCOME [5], CANVAS [6] and DECLARE [7] showed that the sodium-glucose cotransporter 2 inhibitors (SGLT2i’s) empagliflozin, canagliflozin and dapagliflozin reduced either composite death from cardiovascular causes and/or hospitalization for heart failure or death from any cause in patients with T2DM.

Sodium-glucose cotransporter 2 inhibitors are a class of antidiabetic drugs that lower glucose by blocking glucose reabsorption via SGLT2 inhibition in the kidney and thus reduce glucose levels independent of insulin secretion or action [8]. Due to their mode of action SGLTi’s produce a unique shift to catabolic state of metabolism characterized by reduction in HbA1c, increased glucagon/insulin ratio [9–11], weight reduction and increase in circulating ketone levels [12, 13]. It has also been demonstrated that SGLT2i’s induce a shift to utilization of the fasting state substrates fatty acids [13]. To our knowledge increase in ketone utilization in response to SGLT2i treatment has not been demonstrated in vivo or clinically. However, ex vivo rat hearts increase their ketone consumption in response to elevated ketone concentration, indicating that utilization of the substrate is driven by availability [14] and it is thus probable that SGLT2i treatment does increase cardiac ketone utilization. SGLT2i’s do not increase the risk of hypoglycemia since they do not affect counter regulatory mechanisms of glucose homeostasis [15]. In addition SGLT2i induced urinary glucose excursion is strongly blood glucose dependent both in rat [16] and in human [12] and have thus low risk to trigger hypoglycemia. Since SGLT2 inhibitors have positive effects on CV risk factors such as reducing blood pressure, body weight in addition to their HbA1c lowering effect [17, 18] this class of drugs may be of use for intervention in early stages of diabetes/prediabetes [18].

The unexpected positive cardiovascular outcome data from the EMPA-Reg study has triggered interest in the cardiac field for SGLT2 inhibitors and several mechanisms explaining the positive clinical outcome have been proposed [19]. Several studies in preclinical rodent models of established T2DM have shown that SGLT2 inhibitors could improve endothelial function [20–23], reduce myocardial fibrosis, and enhance systolic and diastolic function [24–26]. Recently, SGLT2 inhibitors have also been used in patients showing improvement of peripheral endothelial function [27–30], confirming the translatability of the endothelial function finding [31].

Effects of SGLT2 inhibitors in prediabetic animal models remain less studied and conclusive [32]. Recently, it has been shown that coronary microvascular dysfunction is a powerful predictor for onset of heart failure with preserved ejection fraction (HFpEF) [33] and is highly prevalent phenotype among this patient population [34]. Indeed coronary microvascular dysfunction is also highly predictive of future CV events in patients with [35] and without established T2DM and correlates to index of insulin resistance [36]. We have established a translational approach to study coronary microvascular function from mouse to man by means of non-invasive color Doppler echocardiography assessed coronary flow velocity reserve (CFVR) [37]. In this study, we set out to test the hypothesis that SGLT2 inhibitor could enhance coronary microcirculation.

Diabetes related cardiac complications are a major cause of death and a large burden for the health care systems. It is therefore important to gain understanding which class of anti-diabetic drugs have the most beneficial effects in diabetic patient subgroups and to understand the overall link between SGLT2 inhibition and cardiac outcome. For this, preclinical models that reflect clinical findings need to be established since this kind of research requires interventions which cannot be performed in clinical settings. Hence it is crucial to validate preclinical models reflecting both metabolic and cardiovascular disturbances seen in patients. For this we tested the effects of empagliflozin on metabolism and cardiac function in C57BL/6 J-lep^ob^ mice (ob/ob^−/−^). These mice become insulin resistant due to leptin deficiency and develop severe obesity, moderate hyperglycemia with high insulin release capacity and marked adiposity [38]. Diabetes progression is mild and the ob/ob^−/−^ mice are thus a model for metabolic syndrome and pre/early diabetes [39]. They lack atherosclerosis formation and have been shown to display coronary microvascular dysfunction.

Coronary flow velocity reserve (CFVR) can be assessed using transthoracic color Doppler echocardiography, and in non-atherosclerotic model like the ob/ob^−/−^, it is a measure of microvascular function [40]. The ob/ob^−/−^ mice have been shown to have cardiac contractile dysfunction, characterized by both systolic and diastolic dysfunction [41]. To validate ob/ob^−/−^ mice as a useful model for studying the clinical outcome with SGLT2i’s, we investigated if treatment of ob/ob^−/−^ mice with the SGLT2 inhibitor empagliflozin improves glycemic status, liver function parameters and cardiovascular function by assessing CFVR and FAC (fractional area change is a left ventricular systolic index of contractility [42]). We further aimed to determine microvascular structural and functional changes after empagliflozin treatment and whether the vascular nitric oxide (NO) pathway is involved.

Furthermore, although it is clear that there is an increased incidence of micro- and macrovascular complications [43] as well as worsened cardiovascular outcome [44] in individuals with prediabetes there is a lack of tools for predicting which patients will face cardiovascular complications in the future [43]. To develop a preventative strategy, it is therefore important to study treatment effects with SGLT2i on cardiovascular complications in early stages of metabolic diseases. However, the currently published clinical studies were performed on patients with diabetes and established CV disease. The glucose lowering effect of SGLT2i has been studied both in human and rodents in the healthy and prediabetic state [15, 45] but to our knowledge only little data on the effect of SGLT2i on cardiovascular function in the prediabetic state is available [32]. In analogy to research around SGLT2i effects on cardiovascular function, SGLT2i reduce liver fat as a class effect [46]. In addition to the cardiovascular effect we thus monitored the effect of empagliflozin on liver status in our model for pre/early diabetes as well.

## Methods

**Fig. 1:**
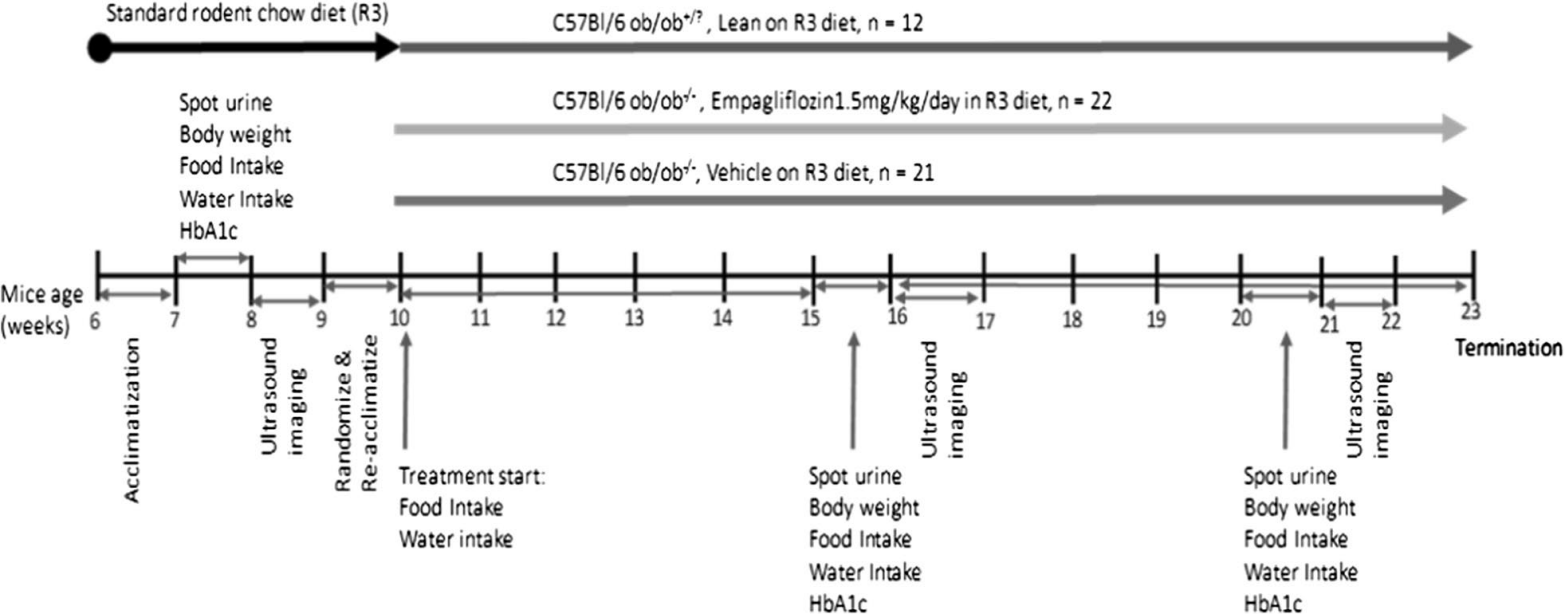
Study design, 55 mice arrived at 6 weeks of age and acclimatized for 1 week prior to entry into the study

In order to investigate the effect of SGLT2 inhibition in a rodent model with mild diabetes and early cardiovascular dysfunction, the study was performed in obese, insulin resistant homozygous male C57BL/6 J-lepob mice [ob/ob^−/−^ mice, treated (n = 22) and untreated n = (21)]. Age matched non-obese male lean littermates (C57BL/6 J ob/ob^+/?^) were used as healthy controls (lean control, n = 12), (Jackson Laboratory, USA). Animals were 6 weeks of age on arrival, had free access to water and standard rodent chow diet (R3, Lantmännen, Stockholm, Sweden) in temperature-controlled facilities with a 12-h light and 12-h dark cycle, at 21–22 °C. Treated ob/ob^−/−^ group received 1.5 mg/kg body weight/day empagliflozin (commercially available empagliflozin was purchased) in R3 pellets (Lantmännen, Stockholm, Sweden). Mice were housed in macrolon polycarbonate cages, with either 4 or 3 animals/cage (ob/ob^−/−^) or one animal/cage (lean) and acclimatized for 1 week before entering the study (Fig. 1). All cages were environmentally enriched with sawdust, nest pads, gnaw sticks and carton. Mice were anesthetized in the imaging laboratory by inhalation of isoflurane gas (2.5%, Abbott Scandinavia, Solna, Sweden) due to its beneficial properties of quick onset and short half-life, thereby minimized anesthesia time.

The ob/ob^−/−^ groups were randomized using body weight and HbA1c. Due to the obvious phenotype differences, blinding was not possible when performing the imaging of ob/ob^−/−^ and lean mice. However, every other lean and ob/ob^−/−^ mouse was examined by ultrasound to minimize time shift. One ob/ob^−/−^ mouse was euthanized at 8 weeks of age because of a breeding defect after been examined by the veterinarian. Blood samples were collected from the tail veins 1 week prior to each ultrasound scanning for HbA1c determination and mice put back in their home cages. At termination, blood was taken for insulin and glucagon in the vena saphena after 4 h fasting in the awake mouse, and from the left ventricle after anesthesia with 5% isoflurane for all other biomarkers. Furthermore, heart and kidneys were collected and weighed. A 4-mm thick slice of heart tissue at the level of mitral valve and the right kidney were collected and immersion fixed in formalin for subsequent histology analysis. All flow velocities (described below) were determined from signals that were stable for at least three consecutive heartbeats and representative of the average of two cardiac cycles. Measurements was performed off-line (Vevo2100 software, Visualsonics Inc., Toronto, Canada) in a blinded fashion.

### Cardiac echocardiography and coronary flow velocity reserve (CFVR) measured using high-resolution ultrasound imaging

Transthoracic echocardiography was performed using non-invasive Doppler ultrasound at 9, 16 and 21 weeks of age in all mice using a high-frequency ultrasound imaging system (Vevo 2100 VisualSonics, Inc., Toronto, Ontario, Canada) with a 40-MHz central frequency transducer to characterize coronary vascular function according to previously established protocols [40]. All mice were anesthetized with Isoba^®^ vet isoflurane (Schering-Plough Ltd., England) in a closed chamber with 3% isoflurane in oxygen for 2 to 5 min until immobile and 1.0–1.5% of isoflurane was used during the examination. Each mouse was placed supine on a heated procedure board with isoflurane initially at 1.5% supplied by a nose cone connected to the anesthesia machine. Chest hair was removed with chemical cream (Veet, Reckitt Benckiser, UK). Isoflurane was reduced to 1% to lower coronary flow to a baseline level and velocity profile in the left coronary artery was monitored for 3 min to ensure stable signal was achieved, after which signals were collected and stored. Isoflurane level was then increased to 2.5% to induce hyperemia, and the velocity profile was monitored for up to 4 min during which time signals were stored for offline analysis of maximum hyperemic response. Isoflurane was reduced back to 1%, and mice allowed stabilize. Detailed protocol using isoflurane to induce hyperemia has been described elsewhere [40].

Measurements of left ventricle dimensions were performed in standard B-mode using images in short axis views at the papillary level, before the CFVR protocol.

FAC was calculated as 100 * ((LV diastolic area − LV systolic area)/LV diastolic area), where LV is the left ventricle.

Left ventricular end-diastolic volume (LVEDV) and left ventricular end-systolic volume (LVESV) was calculated using the Teichholz’s formula:

$${\text{LVEDV}} = \left( { 7*{\text{LVEDD}}^{ 3} } \right)/\left( { 2. 4+ {\text{LVEDD}}} \right),$$

where LVEDD is LV end-diastolic dimension.

$${\text{LVESV}} = \left( { 7*{\text{LVESD}}^{ 3} } \right)/\left( { 2. 4+ {\text{LVESD}}} \right),$$

where LVESD is LV end-systolic dimension.

Stroke volume (SV) was calculated as end-diastolic volume (EDV) − end-systolic volume (ESV).

Cardiac output was calculated as (SV * HR)/1000, where HR is heart rate.

CFVR was calculated as the ratio of peak diastolic flow velocities at baseline obtained using 1% isoflurane, and during hyperemia obtained using 2.5% isoflurane (CFVR = hyperemic coronary flow velocity/basal coronary flow velocity).

Heart rate was calculated as average from three consecutive cardiac cycles in Doppler mode, adjacent to the CFVR bridging heart rate and CFVR in time.

### Biomarker analysis

HbA1c, urine glucose, albumin and creatinine were measured at about 7 to 8 (baseline), 15 to 16 (5 weeks after intervention) and 20 to 21 (10 weeks after intervention) weeks of age, under non-fasting conditions. Further biomarkers were measured at termination after 4 h fast.

The measurement of HbA1c was performed using blood from tail vein (A1CNow+, Bayer Healthcare, Austria). Urine albumin was measured using a commercial ELISA kit (Cat No E-90AL) from ICL, USA. Urine creatinine was measured using an enzymatic colorimetric method (Kit No A11A01933) from Horiba ABX, France. Urine glucose was analyzed using an enzymatic (hexokinase) colorimetric method (ABX Pentra 400, USA).

#### Biomarkers measured at the terminal endpoint (mice at 23 weeks of age)

Enzymatic colorimetric methods were used to measure total cholesterol [(Kit No A11A01634) from Horiba ABX, France], plasma triglycerides [(Kit No 12146029 triglycerides) from Roche Diagnostics GmbH, Germany] as well as triglycerides in the liver [(Kit No A11A01640) from Horiba ABX, France] following homogenization in isopropanol.

Plasma insulin level was measured with a radioimmunoassay (SRI-13K, Millipore Corporation, USA) on a 1470 Automatic Gamma Counter (PerkinElmer, USA). Glucagon level was measured using sandwich ELISA from Mercodia (Mercodia A/S, Uppsala, Sweden). Alanine aminotransferase (ALAT) was measured using the optimized UV-test according to IFCC (International Federation of Clinical Chemistry) modified method without pyridoxal phosphate (ABX Pentra 400, USA). Beta hydroxybutyrate (nmol/well) was measured using colorimetric probe with an absorbance band at 450 nm (Abcam, USA). N-terminal prohormone of brain natriuretic peptide (NTpro BNP) was measured using sandwich ELISA (Kamiya Biomedical Company, USA).

### LC–MS/MS analysis of l-arginine and asymmetric dimethyl arginine (ADMA) in plasma

l-Arginine, ADMA and SDMA was analyzed using a modified liquid chromatography–tandem isotope dilution mass spectrometry analysis (LC–MS) methodology previously described (Sigma-Aldrich, St. Louis, MO, USA) [40].

### Histopathology and immunohistochemistry

Tissue samples were immersion fixed in buffered formaldehyde solution, dehydrated and embedded in paraffin wax. Histological staining was performed on 4 μm thick sections. Hematoxylin–eosin staining was used to evaluate general morphology and for steatosis scoring, Picro sirius red (PS) for the evaluation of fibrosis. Histopathological assessment of fibrosis and liver steatosis was carried out using semi-quantitative scoring where 0 indicates no change and 3 a severe change [47].

CD31 staining on the heart tissue to detect endothelial cells as markers for vessels was performed using anti-CD31 antibody (1:25, #ab28364, Abcam) on the Ventana semi-automated staining machine (Ventana Medical Systems, Inc., USA). Slides were deparaffinized at 69 °C and then underwent antigen retrieval in cell conditioning media at 95 °C. After a blocking step anti-CD31 antibody was applied unto the slides, and incubated for 60 min at 37 °C. The CD31 signal was detected using OmniMap Anti Rb HRP, followed by Discovery DAB CM reagent to give a red color. Finally, sections were counter stained and slides were removed from Ventana and mounted manually.

To enable analysis, stained tissue sections were digitized using a Zeiss Mirax slide scanner (3D Histech, Budapest, Hungary) to image 100% of each section. The resulting virtual slides were imported into BioPix iQ (version 3.3.4, BioPix AB, Gothenburg, Sweden) for CD31 analysis. Analytical tissue sections were selected using the scanned region containing tissue only and resampled at 3.51 mega pixels. The CD31 positive endothelial cells were identified with the computerized color marker which was carefully determined using hue saturation and brightness. The same setting was used for all sections. The outer regions of the tissue, artefacts and large vessels (≥ 200 µm^2^) were identified and excluded from overall vessel analysis. The results are reported as the mean area fraction of the identified vessels compared to the total tissue area examined.

### Statistics

Before analysis, we assumed an increased difference due to time in bodyweight, HbA1c, coronary hyperemic flow velocity reserve (CFVR) and FAC between strains and effect of treatment. Therefore, Anova with 5%-significance was used to compare changes between lean controls, treated and vehicle group at 5 and 10 weeks after intervention. The non-parametric Mann–Whitney U test was used to compare the cardiac vessel density and t-test was used for semi-quantitative values.

## Results

To confirm target engagement, we measured urine excretion at all time points (Fig. 3b). In addition to the increased urine glucose excretion we also observed increased urine volumes (data not shown).

### Glycemic status and weight

**Fig. 2:**
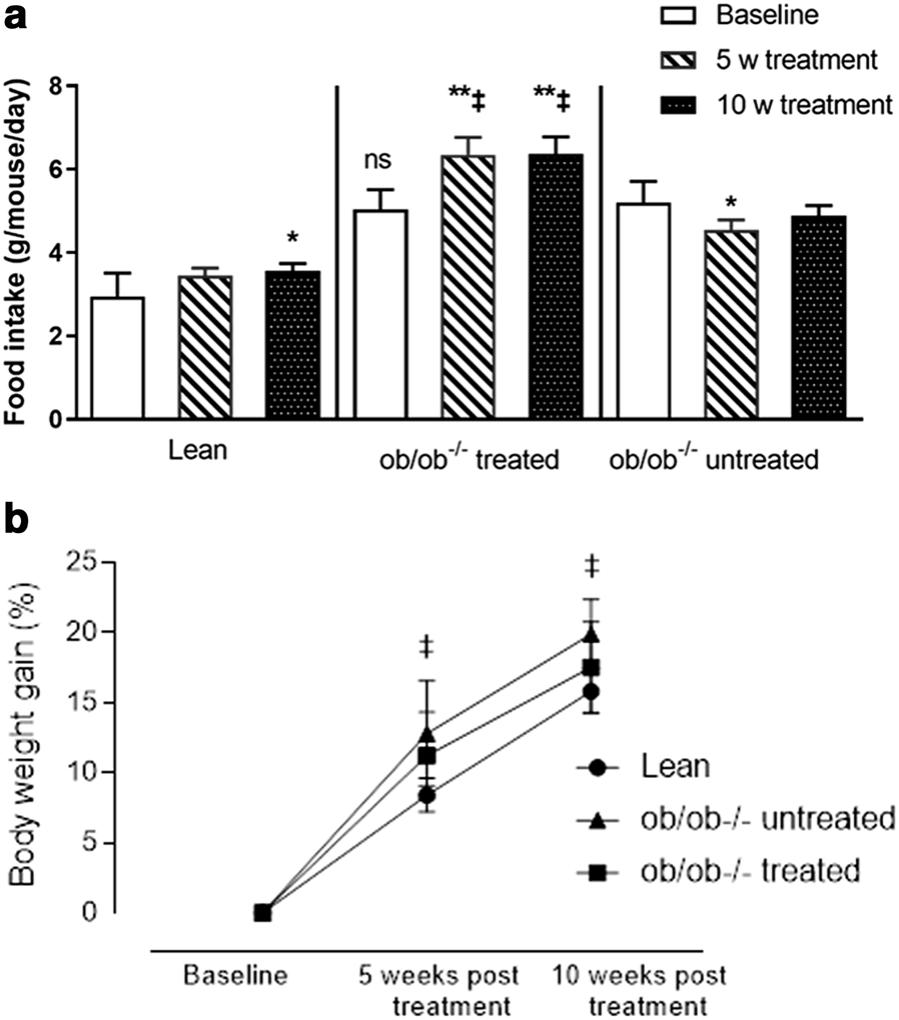
Food intake (**a**) and body weight gain (**b**) in lean and ob/ob^−/−^ with and without treatment at baseline and during empagliflozin treatment. Data were analyzed using Anova with Tukey’s multiple comparisons test and are presented as mean with standard deviation. *p < 0.05 and **p < 0.01 for 5 and 10 weeks compared with baseline, and ^‡^p ≤ 0.05 for ob/ob^−/−^ treated vs. untreated group

As expected, body weight was higher in the ob/ob^−/−^ groups compared with the lean controls at all three time points. The relative increase in body weight was reduced in empagliflozin treated animals compared to untreated ob/ob^−/−^ mice (Fig. 2b) as has been seen in clinical studies. There was no significant difference in wet heart weight; however, treated ob/ob^−/−^ group had a higher kidney wet weight when compared with lean controls and the untreated ob/ob^−/−^ group (Table 1). Upon onset of empagliflozin treatment, the animals increased their food intake at week 5 and 10 compared to baseline and compared to untreated ob/ob^−/−^ animals at the same time points (Fig. 2a). The ob/ob^−/−^ groups had a higher water intake at all time points compared with the lean controls (Table 1). Furthermore, the treated group had a higher water intake at 5 and 10 weeks after intervention compared with the untreated group.

**Table 1:** Weight, water intake and urine albumin data in lean controls and leptin-deficient (ob/ob^−/−^) mice

	Weight			Water intake (ml/mouse/day)	Urine albumin (µg/ml)
	Body (g)	Heart (g)	Kidney (g)		
Lean (n = 12)					
BL	28 ± 1	n.d	n.d	6.7 ± 0.6	9.5 ± 3.0 (n = 2)
5 weeks	30 ± 1	n.d	n.d	5.0 ± 1.2	7.1 ± 2.0 (n = 3)
10 weeks	33 ± 2	0.18 ± 0.04	0.21 ± 0.01	5.4 ± 0.7	7.6 ± 1.4 (n = 6)
ob/ob^−/−^ untreated (n = 21)					
BL	47 ± 4**	n.d	n.d	8.2 ± 2.2*	22.7 ± 5.1 (n = 16)
5 weeks	53 ± 6**	n.d	n.d	9.0 ± 4.0*	15.1 ± 3.1 (n = 19)
10 weeks	60 ± 6**	0.18 ± 0.02	0.22 ± 0.03	8.9 ± 3.8**	10.95 ± 1.8 (n = 12)
ob/ob^−/−^ treated (n = 22)					
BL	48 ± 3**	n.d	n.d	8.1 ± 1.0*	33.0 ± 4.1 (n = 16)
5 weeks	54 ± 3**	n.d	n.d	13.5 ± 1.5*^‡^	7.2 ± 1.1 (n = 9)
10 weeks	58 ± 3**	0.20 ± 0.03	0.24 ± 0.04*^‡^	14.4 ± 2.8**^‡^	5.2 ± 0.3 (n = 5)

To monitor the progression of obesity, body weight was studied over time, and as a measure of animal health, water intake was recorded. Wet heart and kidney weight were also recorded at the end of the treatment period. Statistical significance within each time point by one-way ANOVA and Tukey’s post hoc. Values are presented as mean ± SD for parametric data and mean ± SEM for non-parametric data, where *p < 0.05 and **p < 0.01 and ^‡^p ≤ 0.05 for ob/ob^−/−^ treated and untreated group

**Fig. 3:**
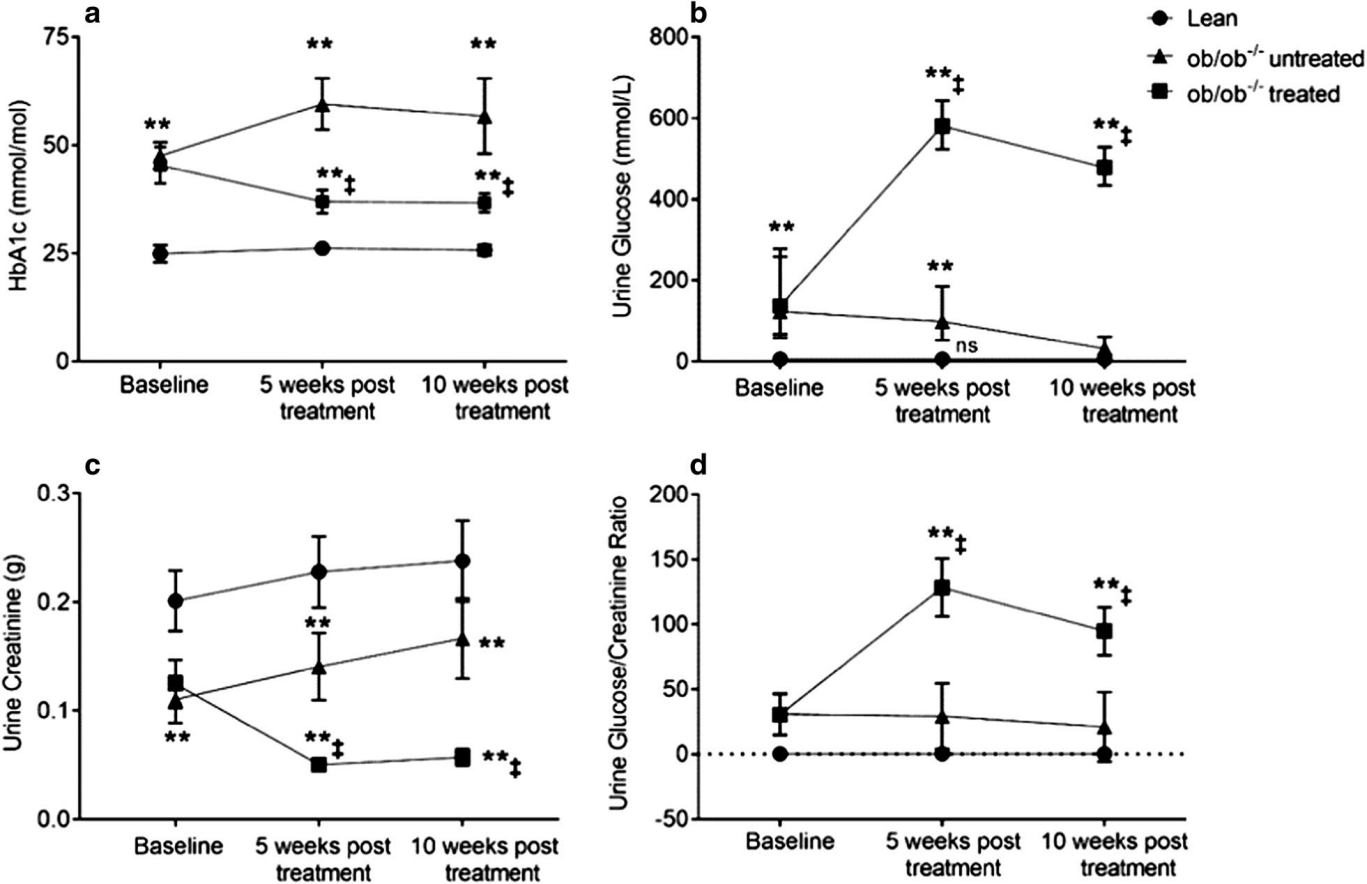
Analysis of **a** HbA1c **b** urine glucose levels, **c** urine creatine and **d** urine glucose/creatinine during the study. In the  treated group (n = 22) HbA1c decreased, increased in ob/ob^−/−^ untreated group (n = 21), and did not change in lean controls (n = 12) compared with baseline levels. Data were analyzed using repeated measure Anova with Tukey’s multiple comparisons test and are presented as mean with 95% confidence interval. *p < 0.05 and **p < 0.01 represents significant difference between lean controls and ob/ob^−/−^ groups. ^‡^p ≤ 0.05 for ob/ob^−/−^ treated and untreated group

HbA1c, an indicator for medium term glucose control was at baseline ~ 2 times higher in both ob/ob^−/−^ groups compared to control. There were no differences between the ob/ob^−/−^ groups at baseline. The empagliflozin treated animals experienced a highly significant reduction in HbA1c compared to untreated animals both at 5 and 10 weeks treatment (Fig. 3a). Taken together, these data indicate that empagliflozin reduced glycemia, promoted caloric loss via glucose excretion and reduced body weight gain.

### Urine kidney biomarkers

Urine albumin (µg/ml) levels were mostly undetectable in the lean group at baseline mean ± SEM (n = 2/12, 9.5 ± 3.0), detectable in both ob/ob^−/−^ groups (untreated n = 16/22, 22.7 ± 5.1, treated n = 16/21, 33.0 ± 4.1). At 5 weeks, fewer lean mice had detectable albumin levels (n = 3, 7.1 ± 2.0) compared with the ob/ob^−/−^ untreated (n = 19, 15.1 ± 3.1) and fewer ob/ob^−/−^ treated compared with untreated (n = 9, 7.2 ± 1.1). At 10 weeks, half of the lean group had detectable albumin levels (n = 6, 7.6 ± 1.4), about half of the untreated group (n = 12, 11.0 ± 1.8) and fewer in the treated group (n = 5, 5.2 ± 0.3). Albumin levels were significantly higher in ob/ob^−/−^ untreated compared with treated group at 5 weeks (p = 0.0424) and at 10 weeks (0.0186) (Table 1).

Urine creatinine (g) was significantly higher in the lean controls compared with the treated and untreated group at all time points. At baseline, both treated and untreated ob/ob^−/−^ groups had similar creatinine levels; however, at 5 and 10 weeks after treatment start urine creatinine was significantly lower in the treated compared with the untreated group (Fig. 3c).

Urine glucose and urine glucose/creatinine ratio (Fig. 3b, d) were significantly higher in the ob/ob^−/−^ groups at baseline compared with the lean controls; however, there were no differences between the ob/ob^−/−^ groups at baseline. Already at 5 weeks after start of empagliflozin treatment, urine glucose and glucose/creatinine ratio were increased more than threefold in ob/ob^−/−^ and these increase were maintained at 10 weeks intervention.

### Coronary and cardiac function

To assess cardiac and coronary artery function during the progression of insulin resistance in non-atherosclerotic ob/ob^−/−^ mice and the effect of SGLT2 inhibition, we performed sequential measurement of CFVR using transthoracic Doppler echocardiography.

Cardiac function was measured by FAC, an ejection phase index of contractile function. 10 weeks of treatment with empagliflozin improved cardiac function in the ob/ob^−/−^ group compared to both vehicle and lean controls (Table 2). At 5 weeks post treatment, treated ob/ob^−/−^ group had a higher FAC compared with lean control, however there was no significant difference between treated and untreated groups of the ob/ob^−/−^. There were no significant differences in FAC between the groups at baseline.

**Table 2:** Echocardiographic data in lean controls and leptin-deficient (ob/ob) treated and untreated mice

	Heart rate (bpm)	Systolic area (mm^2^)	Diastolic area (mm^2^)	FAC (%)	LVEDV (mm^3^)	LVESV (mm^3^)	SV (µl)	CO (µl/min)
Lean (n = 12)								
BL	348 ± 46	5.7 ± 2.4	12.1 ± 2.5	55 ± 12	65.5 ± 17	25.8 ± 14.5	39.7 ± 6.0	13.8 ± 2.1
5 weeks	333 ± 58	8.7 ± 1.9	14.2 ± 1.8	41 ± 8	78.4 ± 14.4	43.6 ± 13.0	34.8 ± 7.3	11.5 ± 2.7
10 weeks	385 ± 45	7.5 ± 2.3	13.7 ± 2.7	46 ± 8	76.8 ± 16.4	35.8 ± 14.0	41.0 ± 6.1	15.7 ± 2.9
ob/ob^−/−^ untreated (n = 21)								
BL	381 ± 44	6.7 ± 1.5	12.7 ± 1.3	48 ± 8	71.2 ± 8.5	33.1 ± 8.6	38.1 ± 5.5	14.8 ± 2.8
5 weeks	358 ± 37	7.7 ± 1.6	14.4 ± 1.7	47 ± 7	82.5 ± 11.8	39.1 ± 9.6	43.4 ± 8.2*	15.0 ± 4.7*
10 weeks	388 ± 43	7.9 ± 1.6	15.4 ± 1.8	49 ± 7	88.8 ± 12.5*	40.1 ± 9.7	48.7 ± 6.8*	19.0 ± 3.1
ob/ob^−/−^ treated (n = 22)								
BL	402 ± 56*	6.5 ± 1.3	12.6 ± 1.5	49 ± 6	70.4 ± 10.2	31.5 ± 7.6	39.0 ± 5.3	15.6 ± 2.5
5 weeks	389 ± 41*	6.6 ± 1.7*	13.1 ± 1.7	50 ± 8*	73.5 ± 11.7	32.5 ± 10.2*	41.0 ± 4.7*	15.9 ± 2.3*
10 weeks	421 ± 39*^‡^	6.3 ± 1.4*^‡^	14.6 ± 1.5	57 ± 8*^‡^	83.3 ± 10.4	30.6 ± 8.3^‡^	52.7 ± 9.6*	22.3 ± 5.0*^‡^

Cardiac function was studied in lean and leptin-deficient (ob/ob^−/−^) treated and untreated mice over time by non-invasive transthoracic ultrasound. Heart rate, short axis systolic and diastolic area, and fractional area change (FAC) was recorded. Statistical significance between groups was tested using Anova, and where there were differences Turkey’s multiple comparison test was carried out. Values are presented as mean ± SD, where *p < 0.05 and **p < 0.01 for significant difference between lean controls and ob/ob^−/−^ groups, ^‡^p ≤ 0.05 for ob/ob^−/−^ treated and untreated group. BL = baseline, mice at 9 weeks of age, 5 weeks = 5 weeks after intervention start, mice at 16 weeks of age, 10 weeks = 10 weeks after intervention start, mice at 21 weeks of age, LVEDV = left ventricular end-diastolic volume, LVESV = left ventricular end-systolic volume, SV = stroke volume, CO = cardiac output

**Fig. 4:**
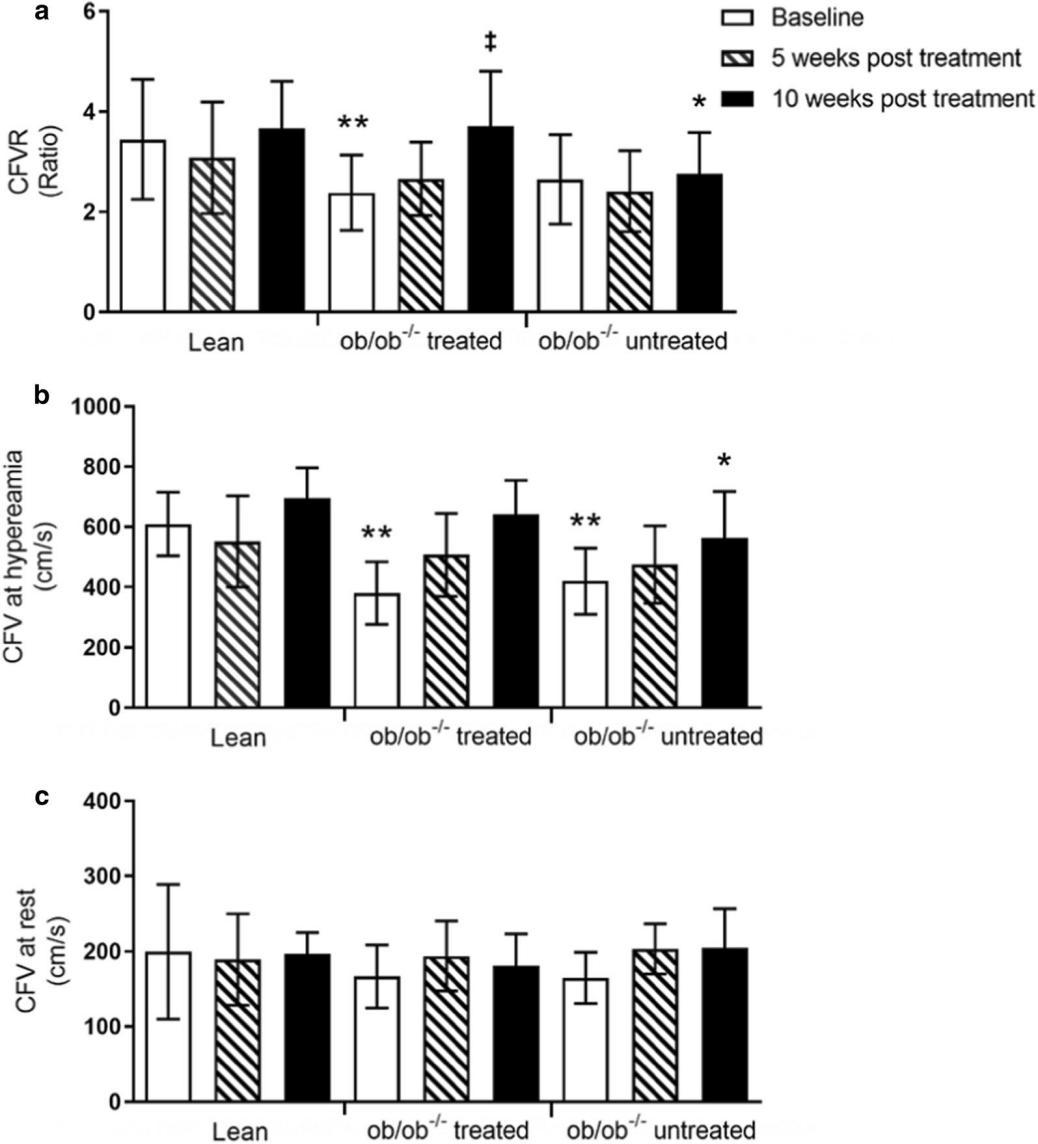
Coronary flow was studied over time in lean, ob/ob^−/−^ treated and untreated mice using non-invasive transthoracic ultrasound. Coronary flow velocity reserve (CFVR) **a** was calculated as the ratio of **b** coronary hyperemic and **c** basal flow velocities. Statistical significance between groups was tested using Anova, and where there were differences Turkey’s multiple comparison test was carried out. Values are presented as mean with standard deviation, where *p < 0.05 and **p < 0.01 for significant difference between lean controls and ob/ob^−/−^ groups. ^‡^p ≤ 0.05 for ob/ob^−/−^ treated and untreated group

Coronary vascular function as measured by CFVR was different at baseline between groups, and this was due to the ob/ob^−/−^ groups having reduced hyperemic flow compared to lean mice. After 5 weeks intervention, there were no detectable differences between groups. At 10 weeks intervention, there was a significant difference between groups, and this was attributable to the untreated ob/ob^−/−^ group having a lower CFVR compared with lean controls and the treated ob/ob^−/−^ group (Fig. 4a). Hyperemic flow (Fig. 4b) after 10 weeks treatment was lower in the vehicle group compared with lean control, and a trend towards having lower values compared with treated ob/ob^−/−^ group. Basal flow velocity was not different between groups at any of the time points (Fig. 4c).

Heart rate was significantly different between groups at all time points, at baseline and 5 weeks post treatment, this was due to the treated ob/ob^−/−^ group having a higher heart rate compared with the lean control group. At 10 weeks intervention the treated ob/ob^−/−^ group had a higher heart rate compared with the untreated ob/ob^−/−^ group, but not when compared to the lean controls (Table 2).

### Fasting biomarkers at termination

#### Cardiac biomarker

There was a trend for N-terminal pro b-type natriuretic peptide to be lower in the treated ob/ob^−/−^ group compared to the untreated, although it did not reach statistical significance (p = 0.0891) (Table 3).

**Table 3:** Plasma biomarkers at termination after 4 h fast in lean controls, ob/ob^−/−^ treated and untreated mice

	Lean	ob/ob^−/−^ untreated	ob/ob^−/−^ treated
Cholesterol (mM)	2.6 ± 0.3	5.0 ± 0.9**	3.8 ± 0.3**^‡^
Triglycerides (mM)	0.5 ± 0.1	0.6 ± 0.3	0.5 ± 0.2
ALAT (µkat/l)	0.47 ± 0.13	8.81 ± 2.85**	2.63 ± 0.77**^‡^
Glucagon (pmol/l)	12.5 ± 9.5	16.4 ± 14.1	17.2 ± 5.4**
Insulin (ng/ml)	0.3 ± 0.2	7.3 ± 2.6**	6.5 ± 3.2**
Glucagon/insulin ratio	48.7 ± 50.1	1.7 ± 0.5**	3.0 ± 1.5**^‡^
Beta hydroxybutyrate (µM)	313 ± 97	133 ± 44	489 ± 56*^‡^
NT-proBNP (pg/ml)	–	8957 ± 1923	8216 ± 1462
l-Arginine (μM)	69 ± 13	26 ± 21**	41 ± 28**^‡^
ADMA (μM)	0.69 ± 0.07	0.79 ± 0.13*	0.80 ± 0.13*
l-Arginine/ADMA ratio	101 ± 16	33 ± 30**	51 ± 34**^‡^
SDMA (μM)	0.20 ± 0.02	0.16 ± 0.03**	0.16 ± 0.02**

Functional endothelial data, measures of diabetes, kidney and liver status were studied. Statistical differences between groups was tested using Anova for parametric parameters (SDMA, insulin, and insulin/glucagon ratio) and Kruskal–Wallis test for non-parametric parameters (l-arginine, ADMA, l-arginine/ADMA ratio, glucagon, ALAT, cholesterol, triglycerides and beta hydroxybutyrate). Values are presented as mean ± SD for parametric data and mean ± SEM for non-parametric data, where *p < 0.05 and **p < 0.01 for between group analysis, and ^‡^p ≤ 0.05 for ob/ob^−/−^ treated and untreated group

#### Lipid biomarkers

**Fig. 5:**
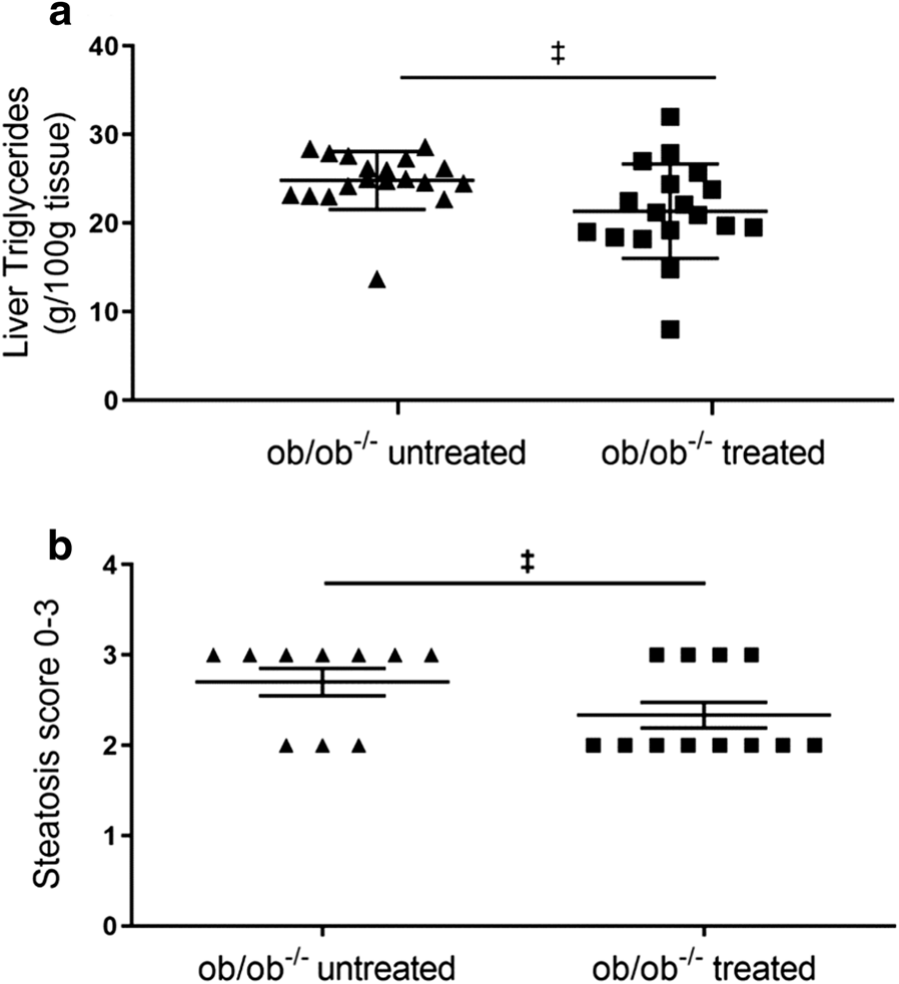
Triglyceride levels and steatotic count. Triglycerides levels in liver tissue in treated (n = 18) compared to untreated (n = 19) ob/ob^−/−^ group (**a**). Steatosis score in treated (n = 12) compared to untreated (n = 10) ob/ob^−/−^ group (**b**). Data were analyzed using two-tailed Mann–Whitney test for liver triglycerides and t-test for steatosis score and are presented as mean with standard deviation. ^‡^p ≤ 0.05 represents significant difference between ob/ob^−/−^ treated and untreated group

To investigate the effect of SGLT2 inhibition on lipid profile we measured fasting plasma cholesterol, plasma triglyceride and liver tissue triglyceride in the ob/ob^−/−^ treated and untreated groups. Both ob/ob^−/−^ groups had higher cholesterol compared with the lean control, furthermore treated ob/ob^−/−^ group had lower cholesterol compared with untreated group (Table 3). No difference was observed with plasma triglyceride (Table 3); however, liver tissue triglyceride was lower in the treated ob/ob^−/−^ group compared with the untreated (Fig. 5). Furthermore, ALAT an enzyme, which indicates liver health, although higher in both ob/ob^−/−^ groups compared with lean controls, was significantly lower in treated ob/ob^−/−^ group compared with untreated.

#### Metabolic profile

As expected at 23 weeks of age after 4 h fasting, insulin levels in the ob/ob^−/−^ groups were increased compared to lean mice. The elevated insulin levels in both ob/ob^−/−^ groups at the end of the study in combination with increased HbA1c compared to lean animals are indicative of insulin resistance typical for ob/ob^−/−^ mice. Glucagon was higher in the ob/ob^−/−^ groups compared with lean mice, and there was a trend for glucagon to be higher in the treated ob/ob^−/−^ mice compared with the untreated mice (Table 3). Glucagon/insulin ratio was lower in the ob/ob^−/−^ groups compared with the lean mice, and higher in the treated ob/ob^−/−^ mice compared with the untreated mice (Table 3). The treated ob/ob^−/−^ mice had almost 4 times higher beta-hydroxybutyrate levels compared with untreated ob/ob^−/−^ mice which matches clinical studies reporting elevated ketone levels in patients who received SGLT2i treatment. Ketone levels in untreated ob/ob^−/−^ mice were about 40% of what was seen in lean animals.

To investigate the nitric oxide pathway, we measured l-arginine and ADMA in plasma. We found significantly lower plasma levels of l-arginine (Table 3) in ob/ob^−/−^ mice compared to lean controls, and a higher level of ADMA indicating dysfunctional nitric oxide pathway. The l-arginine/ADMA ratio was significantly lower in ob/ob^−/−^ mice compared to lean mice (Table 3), and furthermore l-arginine/ADMA ratio was higher in treated ob/ob^−/−^ mice compared with untreated indicating an improved nitric oxide pathway.

#### Histopathology and immunohistochemistry

**Fig. 6:**
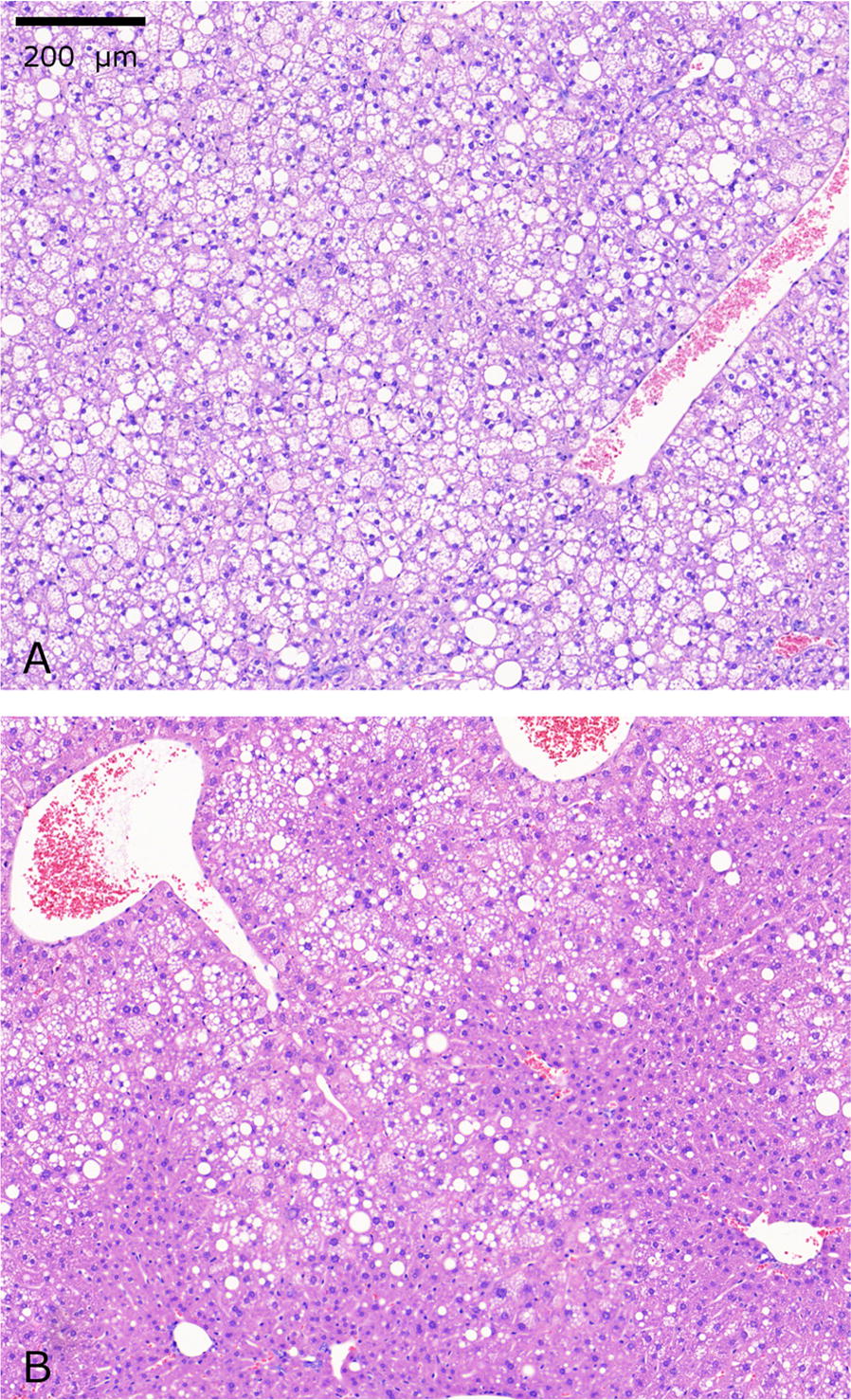
Representative images of liver sections stained with hematoxylin–eosin showing patches of vacuolated hepatocytes. Liver section from an untreated ob/ob^−/−^ mouse with highly vacuolated hepatocytes and a high steatosis score (**a**). Liver section from a treated ob/ob^−/−^ mouse with reduced steatosis score (**b**). The scale bar equals 200 µm in each figure

There were no clear changes in the fibrosis of the heart, kidney or liver between the treated and untreated ob/ob^−/−^ mice, while there was a statistical difference in liver steatosis score between treated and untreated mice (Fig. 5). Patches of vacuolated hepatocytes were detected in the liver indicative of steatosis (Fig. 6).

**Fig. 7:**
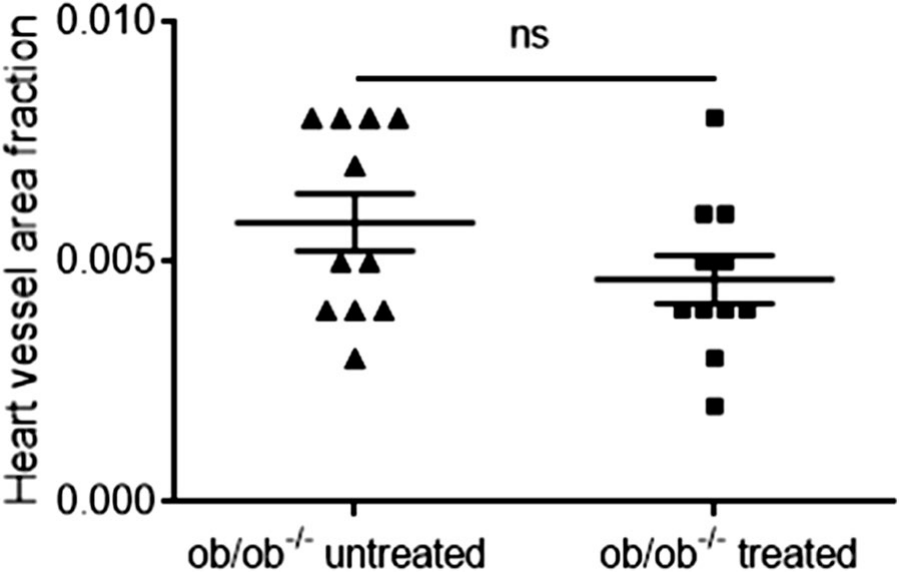
Heart vessel area fraction was not significantly different between treated and untreated ob/ob^−/−^ mice (n = 11 per group). Data analyzed using two-tailed Mann–Whitney test, and are presented as mean with standard deviation

To study structural vascular changes in the heart, we measured cardiac vessel area fraction. When comparing cardiac vessel area fraction in the untreated ob/ob^−/−^ (0.0058 ± 0.0020) and treated ob/ob^−/−^ mice (0.0046 ± 0.0016), no statistically significant difference was detected (Fig. 7).

## Discussion

We demonstrate in the present study that the SGLT2 inhibitor empagliflozin improved contractile function of the heart, liver function, lipid profile, increased l-arginine/ADMA ratio as an indicator of increased nitric oxide production and improved glycemic status, in ob/ob^−/−^ mice.

### SGLT2 inhibition improves systolic contractility and coronary microvascular function which might explain improved clinical cardiovascular outcome seen with SGLT2i’s

The ob/ob^−/−^ mice heart has been shown to be metabolically inefficient [48], with reduced myocardial efficiency at an early age [49], impaired cardiac contractile function and left ventricular hypertrophy [50]. We detected a statistically significant increase in fractional area change (FAC), a systolic contractility index [42] and we observed significantly improved coronary microvascular function after 10 weeks of treatment in the ob/ob^−/−^ mice. Based on the knowledge from humans that coronary flow reserve and cardiac systolic function are powerful predictors of cardiovascular outcome [51, 52], we believe that the current findings are of clinical relevance and provide direct physiological evidences of SGLT2 inhibitors CV benefit [5].

In a none atherosclerotic model like the one  used in this study CFVR, which usually is a measure for both coronary macro- and microvascular function, becomes a direct indicator of microvascular function [40]. This has been further demonstrated by us recently in patients with HFpEF without epicardial coronary artery diseases, where a correlation between CFVR and peripheral endothelial function was observed [34]. Coronary microvascular function is determined in part by both endothelial dependent [53] and independent mechanisms [37, 53]; improved l-arginine/ADMA ratio is proposed to be involved in the improved CFVR. Consistent with our findings, a recent study in db/db mouse showed an improvement in cardiac diastolic function upon empagliflozin treatment [54]. Furthermore, in diabetic Zucker rats, SGLT2 inhibition using dapagliflozin showed improved cardiac function [55], albeit these are advanced models of diabetes. Furthermore, in a non-diabetic pressure overload-induced heart failure mouse model, empagliflozin blunts the decline in cardiac function independent of diabetes [56]. Diabetes, in particular insulin resistance is proposed to play a role in the development of heart failure and cardiac dysfunction [57]. Obese individuals without diabetes but with insulin resistance syndrome manifest similar increased risk for CV disease when compared with T2DM patients [58], and in individuals with heart failure, FAC has been shown to be reduced [59]. Even though coronary flow reserve and systolic function independently of each other and synergistically predict CV mortality [52], improved CFVR may partly contribute to the improved FAC, given that CFVR may reflect improved energy utilization by decrease baseline energy need and improved flow/energy reserve.

### SGLT2 inhibition raises l-arginine/ADMA ratio possibly causing improved coronary microvascular function via NO-dependent improvement of endothelial function

It has previously been shown that plasma levels of l-arginine in ob/ob^−/−^ mice is lower compared to lean mice, indicating dysfunctional nitric oxide pathway, while no difference was seen in ADMA levels [40]. We reproduced these findings in our study, and further showed that l-arginine was higher in empagliflozin treated ob/ob^−/−^ mice compared to untreated, resulting in higher l-arginine/ADMA ratio. l-arginine is the substrate for both nitric oxide synthase (NOS), which converts arginine to nitric oxide (NO) and citrulline, as well as for arginase that converts l-arginine to urea and ornithine. ADMA is an endogenously produced inhibitor of NOS, which has a similar structure to arginine and competes with arginine for NOS binding, hereby blocking the formation of NO from arginine by NOS directly [60]. Endogenous arginine synthesis is an inter-organ process that involves citrulline produced in the small intestine which is then transported in the blood and used for arginine synthesis in the kidney [61]. We speculate that the increase in l-arginine with SGLT2 inhibition could be because of increased synthesis in the kidney. The main site of action of SGLT2 inhibitors is the kidney, and the extent of the pleiotropic effects that this class of drugs have been still unknown. It is known that l-arginine is a physiological precursor to the formation of NO in endothelium-dependent vasodilation; and NO has several intracellular effects that lead to vasorelaxation, endothelial regeneration, inhibition of leukocyte chemotaxis, and platelet adhesion [62]. In the ob/ob^−/−^ mice, decreased cardiac NO production and increased oxidative stress have previously been demonstrated [63]. We therefore speculate that the observed improvement in l-arginine/ADMA ratio in our study could lead to an increase in NO bioavailability, thereby improving coronary and cardiac function. The lack of detectable difference in CD31 staining suggests that the mechanisms involved in the observed improved CV function does not involve a recovered structural capillary rarefaction. The improved l-arginine/ADMA ratio points to NO-dependent improvement of endothelial function as the relevant pathway for improved microvascular function.

### Empagliflozin improves overall liver status and lowers hepatic triglyceride content, an independent marker for diastolic function, and potentially contributes to improved contractility

Plasma alanine aminotransferase, hepatic triglyceride and steatosis scoring were lower in the empagliflozin treated ob/ob^−/−^ compared with the untreated mice. This is consistent with the finding in a mouse model of non-alcoholic steatohepatitis (NASH) and diabetes, where empagliflozin attenuated the development of NASH showing anti-steatotic effects [64]. Furthermore, our data is consistent with that published on the effect of ipragliflozin treatment in ob/ob mice [65]. Hepatic triglyceride content is closely associated with steatosis in NASH as well as with de novo lipogenesis [66], therefore, SGLT2 inhibitors may have potential therapeutic efficacy in T2DM-associated hepatic steatosis. Furthermore, hepatic triglyceride content has been shown to be associated with diastolic function independent of confounding factors including the metabolic syndrome in obese individuals [67]. SGLT2 inhibitors also mediates a metabolic switch from glucose to lipid utilization [68], and given the bi-directional relationship between fatty liver and CV disease [3], it is plausible that the reduction in hepatic triglyceride we observed in our study contributed in part to the improvement in cardiac contractile function. We observed reduced total-cholesterol and no changes in plasma triglyceride with empagliflozin treatment, which is partly different to observations from clinical trials, where no changes were observed in total-cholesterol and plasma triglyceride [69]. It is plausible that these differences are because of species differences in lipid profiles.

### Overall effects of empagliflozin on HbA1c, urinary glucose excretion, body weight and water intake mimic clinical findings with SGLT2 inhibitors

As expected, urine glucose excretion was elevated in the treated group in line with the mode of action of SGLT2 inhibitors. Food and water intake for the lean mice were comparable to published data [70]. Food intake for treated ob/ob^−/−^ mice was higher than in the untreated group and body weight was not different prior to treatment start. In line with clinical studies, we found that body weight increase was lower in the treated mice compared to the untreated. Furthermore, consistent with published data [71], we found that empagliflozin enhanced water intake, possibly to compensate for the urinary glucose, and fluid loss, thereby maintaining fluid homeostasis and stabilizing body weight. We showed that urine albumin and creatinine were reduced in the treated groups. It is well established that increased urine albumin is an independent risk factor in kidney and cardiovascular disease [72], and the reduction of albuminuria has been shown to reduce cardiovascular events [73]. The depletion of creatinine however could be caused by various factors such as changes in muscle mass, creatinine reabsorption, and cell leakage [74], therefore we are unable to ascertain the exact cause or importance of the reduced creatinine we observed.

### SGLT2 inhibition shifts metabolism to a more catabolic state contributing to improved cardiac function

Positive association between higher HbA1c levels and increased CV event rates has been shown in several studies, highlighting the need for glycemic control to prevent or delay the onset of CV morbidity and mortality [75]. In line with published studies using rodent models [15], and clinical studies [5], glycemic status as measured by HbA1c was significantly improved in the treated group. T2DM is associated with increased basal plasma glucagon levels, and it has been shown that in individuals with T2DM, SGLT2 inhibition further increases plasma glucagon. In addition it has been demonstrated that SGLT2 inhibition increases glucagon secretion from pancreatic islets [45, 76]. In our study, we were unable to detect differences in fasting plasma glucagon levels between treated and untreated ob/ob^−/−^ mice, however, the glucagon/insulin ratio was significantly higher in treated compared with untreated ob/ob^−/−^ mice. Consistent with findings in clinical studies [77], and animal study [78], empagliflozin treatment caused a significant increase in fasting plasma ketone concentration in ob/ob^−/−^ mice. The mechanisms involved in increased circulating ketone body levels with SGLT2 inhibition are not fully understood, however it is proposed that it may relate to a decrease in renal clearance of ketone bodies [79]. In addition, it is well established that elevated glucagon/insulin ratios favor ketone production and release from the liver. Furthermore, we speculate that under this condition of increased circulating ketone levels, the heart and other organs take up β-hydroxybutyrate and this is oxidized in preference to fatty acids, resulting in improved cardiac efficiency [80].

### Study limitations

In the current study we demonstrated improved coronary flow reserve in an animal model without macrovascular complications. In humans, CFVR reflects both macro- and microvascular disease [37]. Given the anti-atherosclerotic effects also may translate into man [81], the potential magnitude of change of CFVR may be even higher in humans during longer term treatment than what is demonstrated in the current study. Further, while we showed that NO-pathway maybe involved in the improved CFVR, in humans with complex disease background, e.g. hypertension, dyslipidemia, as well as environmental risk factors, the translatability of this mechanistic finding needs be to confirmed. Finally, despite the human relevant CFVR protocol we have developed and applied in the current study, this important physiological measure is still assessed during anesthesia. Even though all animals were handled in a similar way, cautions still need to be taken when extrapolating the finding into humans.

## Conclusion

To our knowledge, there are currently no published clinical studies showing that SGLT2 inhibitors improves coronary microvascular and systolic function in early stages of diabetes/prediabetes. We are however aware that there is a clinical study registered to investigate the effect of dapagliflozin in T2DM or pre-diabetes and preserved ejection fraction heart failure that is scheduled to complete in March 2019 (NCT03030235) [82]. Our data serves as preclinical evidence for this clinical study, in which a positive outcome could provide a paradigm shift in the way individuals with prediabetes are treated in the future. Our study demonstrated that in a mouse model with insulin resistant phenotype, moderate hyperglycemia and coronary vascular dysfunction, SGLT2 inhibition using empagliflozin is efficacious in the improvement of coronary and cardiac dysfunction as well as liver function, possibly because of improved endothelial function.

## Abbreviations

ADMA: asymmetric dimethyl arginine
ALAT: alanine aminotransferase
CV: cardiovascular
CFVR: coronary flow velocity reserve
FAC: fractional area change
LV: left ventricle
NO: nitric oxide
NASH: non-alcoholic steatohepatitis
SGLT2i: sodium-glucose cotransporter 2 inhibitor
T2DM: type 2 diabetes mellitus
